# Nanosecond Laser–Fabricated Monolayer of Gold Nanoparticles on ITO for Bioelectrocatalysis

**DOI:** 10.3389/fchem.2020.00431

**Published:** 2020-06-04

**Authors:** Vivek Pratap Hitaishi, Ievgen Mazurenko, Anjali Vengasseril Murali, Anne de Poulpiquet, Gaëlle Coustillier, Philippe Delaporte, Elisabeth Lojou

**Affiliations:** ^1^Aix Marseille Univ, CNRS, BIP, Bioénergétique et Ingénierie des Protéines, UMR 7281, Marseille, France; ^2^Aix Marseille Univ, CNRS, LP3, UMR 7341, Parc Scientifique et Technologique de Luminy, Marseille, France

**Keywords:** laser, nanoparticles, electrode nanostructuration, enzymes, catalysis, self-assembled-monolayers, electrochemistry

## Abstract

Redox enzymes can be envisioned as biocatalysts in various electrocatalytic-based devices. Among factors that play roles in bioelectrochemistry limitations, the effect of enzyme-enzyme neighboring interaction on electrocatalysis has rarely been investigated, although critical *in vivo*. We report in this work an in-depth study of gold nanoparticles prepared by laser ablation in the ultimate goal of determining the relationship between activity and enzyme density on electrodes. Nanosecond laser interaction with nanometric gold films deposited on indium tin oxide support was used to generate *in situ* gold nanoparticles (AuNPs) free from any stabilizers. A comprehensive analysis of AuNP size and coverage, as well as total geometric surface vs. electroactive surface is provided as a function of the thickness of the treated gold layer. Using microscopy and electrochemistry, the long-term stability of AuNP-based electrodes in the atmosphere and in the electrolyte is demonstrated. AuNPs formed by laser treatment are then modified by thiol chemistry and their electrochemical behavior is tested with a redox probe. Finally, enzyme adsorption and bioelectrocatalysis are evaluated in the case of two enzymes, i.e., the *Myrothecium verrucaria* bilirubin oxidase and the *Thermus thermophilu*s laccase. Behaving differently on charged surfaces, they allow demonstrating the validity of laser treated AuNPs for bioelectrocatalysis.

## Introduction

Redox enzymes are sustainable alternatives to noble metal catalysts or to inorganic catalysts requiring synthesis in organic solvents for various electrocatalytic-based devices. Major challenges, however, are to be solved before such biodevices can enter the market. Among these challenges, long-term stability, and costs linked to enzyme production have to be considered through fundamental studies of enzyme behavior at electrochemical interfaces. Considerable advances have been made during the last 20 years in the development of interfaces able to enhance catalytic currents and the stability of bioelectrodes. Redox enzymes for oxygen reduction reaction (bilirubin oxidases (BOD) and laccase (LAC) in particular) have been widely studied (Mazurenko et al., 2017; Hitaishi et al., 2018a,b). Although some key parameters toward bioelectrode rationalization are now available, further optimization requires the knowledge of relationships between orientation, conformation, and loading of enzymes and electroactivity. In particular, the effect of enzyme-enzyme neighboring interaction on electrocatalysis is an intriguing question. *In vivo*, crowding minimizes protein deformation to unfolded states (Zanetti-Polzi et al., 2014; Kuchler et al., 2016). Does a similar situation occur once an enzyme is immobilized on the electrochemical interface? In a previous work aiming to advance in this knowledge, we highlighted that the full coverage of bilirubin oxidase on gold electrodes does not translate in higher specific activity (Hitaishi et al., 2018a). McArdle et al. (2015) showed an optimum enzyme surface coverage, beyond which the activity decreased, clearly demonstrating the intricate link between electroactivity and enzyme surface coverage. The rigidification of proteins, as well as their aggregation on electrode surfaces, are processes that have been often neglected, but they could influence the percentage of electroactive enzymes (Hitaishi et al., 2018a).

One way to control and tune enzyme surface coverage is the use of patterned surfaces with specific enzyme immobilization. Such surfaces can be obtained via the formation of mixed self-assembled-monolayers (SAMs) based, for example, on thiols carrying different functionalities (Kong et al., 2020). The difficulty here is the partition of the thiol functions on the surface. Controlled assembly of particles on electrode surfaces is another popular functional unit for the modification of electrode surfaces toward enzyme immobilization (Pankratov D. et al., 2014; Kizling et al., 2018). These particles can be of biological origin. As illustrations bacteriophage particles and DNA origami scaffolds were patterned on gold electrodes to study the impact of scaffolding on bioelectrocatalysis (Patel et al., 2017; Ge et al., 2019). Particles can also be metallic, with gold-based nanoparticles (AuNPs) being the most widely used. Many examples in the literature report enhanced bioelectrocatalytic O_2_ reduction thanks to enzymes including LAC and BOD immobilization on electrodes modified by various AuNPs (Pita et al., 2013; Di Bari et al., 2016; Kizling et al., 2018). However, as far as we are aware, very few studies report patterned electrodes based on AuNPs with the aim to control and vary the enzyme coverage for bioelectrocatalysis. Very recently, however, nanosecond laser–treated and heat-sintered gold films were used for ascorbate sensing (Stankevicius et al., 2019) and enzymatic glucose oxidation (Lee et al., 2019), respectively. The latter work especially suggested that inter-enzyme agglomeration is a critical parameter for bioelectrocatalysis that can be overcome by the spatial control of enzyme immobilization.

Laser-material processing is indeed a convenient tool to obtain NP monolayers on the electrode surface (Naser et al., 2019). Laser technology is widely exploited to heat materials over a short period of time and in a spatially confined region of interest on the material surface (Palneedi et al., 2018). The unique interactions of laser radiation with metal surfaces would lead to permanent changes of the material properties in a specific region such as local chemistry and morphology, depending upon the kind of laser-material interaction. The process of AuNP formation by laser ablation can be divided into two major steps. The irradiation of a metal film by a nanosecond pulsed laser first leads to the melting of the metal film. Nanosecond lasers are widely used for the melting step, as under nanosecond laser irradiation, melting is the dominant process (Henley et al., 2005). Once the thin film is molten, hydrodynamic instability may lead to the formation of droplets if the liquid phase poorly wets the substrate. NP formation from the molten metal phase is explained by the spinodal dewetting (Trice et al., 2007). Spinodal dewetting refers to the growth of a fluctuation in thickness. Generally, in thin metal films, it takes place when attractive intermolecular forces overcome the stabilizing effect of the interfacial tension (Seemann et al., 2001). Compared to more classical AuNP suspension prepared via chemical routes, the expected advantages of laser-treated electrodes are multifold. First, laser-obtained NPs are free from any surfactants/stabilizers (Balasubramanian et al., 2010; Alex and Tiwari, 2015), hence their surface chemistry can be tailored as required for biomolecular interactions. Second *in situ* formed laser-NP layers are expected to be more stable and controllable than multilayers obtained through AuNP drop casting (Pankratov D. V. et al., 2014). Laser procedure also avoids the uneasy steps of NP post-anchorage for example by dithiol chemistry. Finally, laser-NP assembly does not require any special storage, making the resulting electrode more promising for disposable bioelectronics.

In this article, we describe nanosecond laser interaction with nanometric gold films deposited on indium tin oxide (ITO) support to generate *in situ* AuNPs (AuNP@ITO). We first discuss the impact on the NP formation of the typical parameters such as the gold film thickness, laser energy, interaction time scale, and pulses. A full analysis of AuNP size and coverage, as well as total surface vs. electroactive surface, is provided as a function of the thickness of the treated gold layer. Using microscopy and electrochemistry, the long-term stability of AuNP@ITO electrodes in the atmosphere and in the electrolyte is demonstrated. Finally, enzyme adsorption and bioelectrocatalysis are evaluated after specific chemical modification of AuNPs. Two multicopper enzymes (MCOs) are particularly studied, i.e., the *Myrothecium verrucaria* BOD and the *Thermus thermophilu*s LAC to demonstrate the validity of AuNP@ITO electrodes for bioelectrocatalysis.

## Experimental

### Materials and Reagents

Ethanol analytical grade 96% (v/v), 6-mercaptohexanoic acid (6-MHA), cysteamine (CYST), sodium acetate (NaAc), acetic acid (CH_3_COOH), sodium hydroxide 97% (NaOH), sodium phosphate (Na_2_HPO_4_/NaH_2_PO_4_), potassium ferricyanide (FeCN), and sulfuric acid 95–98% (H_2_SO_4_) were purchased from Sigma-Aldrich. 100 mM sodium acetate/phosphate buffer solutions were prepared by mixing NaAc, Na_2_HPO_4_/NaH_2_PO_4_, and acetic acid in an appropriate ratio. All solutions were prepared with Milli-Q water (18.2 MΩ cm). Indium-tin oxide (ITO) slides (25 × 75 mm) were purchased from Osilla (osilla.com). BOD from *Myrothecium verrucaria* (*Mv* BOD) was a gift from Amano Enzymes Inc. (Nagoya, Japan). LAC from *Thermus Thermophilus* HB27 (*Tt* LAC) was expressed and purified in our lab according to Hitaishi et al. (2020).

### Electrode Preparation and Characterization

We used commercially available ITO films on glass as a conductive support to further deposit gold films. In order to use the ITO slides as electrodes, roughly 1 × 1 cm^2^ square samples were prepared. The ITO samples were sonicated in ethanol/water (1:1) solution for 15 min and dried under heavy air flux. Then, thin gold films of desired thickness on precleaned ITO were deposited using smart coater (JEOL_781186455) under ~4 Pa pressure, and the sample was named Au-film@ITO.

A PSIA XE-100 atomic force microscope (AFM) was used to measure the thickness of the Au-films on ITO. AFM images were analyzed using the XEP software.

A Quanta-Ray *LAB-190* pulsed (8 ns) Nd:YAG laser operating at 532 nm with maximum energy 550 ± 10% mJ/pulse and 10 Hz repetition rate was used to perform laser irradiation. Laser energy and irradiation modes were modulated by a conjunction of half wave plate plus polarizer, and a pulse generator controls the irradiation mode. Irradiation fluence (mJ/cm^2^) was precisely calculated by defining the spot size of the laser-treated area (~ 1.41 × 1.42 mm^2^) and measuring the incident laser energy. Laser-prepared Au nanoparticles on the ITO sample were named as AuNP@ITO.

AuNP@ITO samples were observed by a JEOL JSM-6390 scanning electron microscope (SEM) with maximal resolution of 3.0 nm (at 30 kV). An accelerating voltage of 10 keV with a small working distance of 10–12 mm was used. Tilted sample imaging has been done using Vega3 Tescan SEM at an accelerating voltage of 20 kV. SEM micrographs were further analyzed using ImageJ software (powered by Fiji) in order to calculate the sizes, densities, projected area (A_projected_), and effective area (A_real_) of the AuNPs on the ITO. In each case, at least 3 images taken at different spots were analyzed and averaged.

### Electrochemical Setup and Bioelectrode Preparation

Electrochemical measurements (Cyclic voltammetry (CV) and chronoamperometry) with AuNP@ITO, Au-film@ITO, or bare ITO electrodes were performed in a standard 3-electrode cell using a potentiostat from Autolab PGSTAT30 controlled by Nova software (Eco Chemie). Hg/Hg_2_SO_4_ and Pt-wire were used as reference and auxiliary electrodes, respectively. All potentials are quoted vs. Ag/AgCl reference electrode by adding 430 mV to the measured potential.

**Scheme 1:**
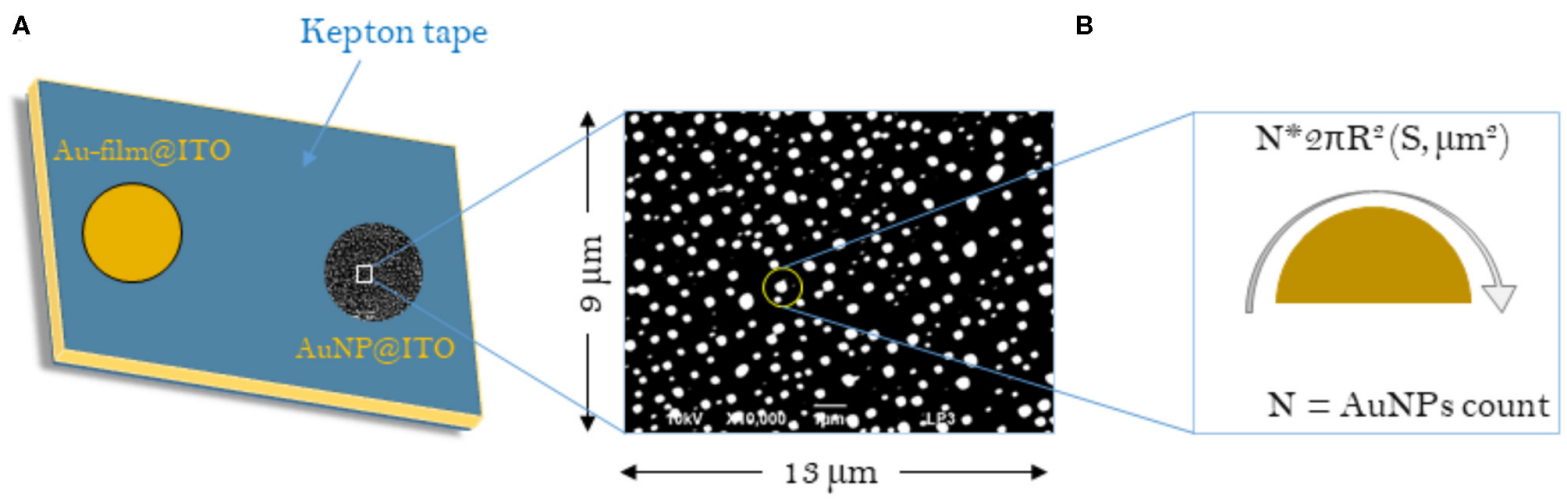
Schematic representations of the samples considered for the calculation of surface areas (S) for Au-film@ITO and AuNP@ITO electrodes. **(A)** Scheme of the Au-film@ITO or AuNP@ITO electrode showing the electro-accessible surface defined by the kepton mask (0.0078 cm^2^). **(B)** Typical SEM image with respective dimensions. The sum of all AuNP surface areas gives the A_real_ for a given SEM image and is then corrected to the total electrode surface (0.0078 cm^2^).

For electrochemistry, an electrode surface of 0.0078 cm^2^ was defined on Au-film@ITO and AuNP@ITO samples with the help of a KEPTON insulating tape (Scheme 1). Au-film@ITO and AuNP@ITO samples were cleaned electrochemically by cycling (10 CV cycles) the potential between 0.2 and 1.5 V vs. Ag/AgCl in 50 mM H_2_SO_4_ at a scan rate of 100 mV·s^−1^. The electroactive area (A_elect_) was calculated from the charge under the CV peak at 0.9 V (peak 3 in **Figure 3**), assuming the charge for gold oxide reduction to be equal to 390 μC cm^−2^ (Trasatti and Petrii, 1991). Finally, self-assembled monolayers (SAMs) were formed by incubating the pretreated electrode in 5 mM ethanolic thiol solutions for 15 min. For enzyme adsorption, unless otherwise indicated, electrodes (whether modified or unmodified) were incubated for 15 min at 4°C with a freshly prepared 20 μM enzyme solution in 100 mM phosphate or an NaAc buffer at the desired pH. The enzyme-modified electrode was then gently washed with the same buffer to remove the loosely adsorbed enzymes and transferred to the electrochemical cell containing 100 mM buffer at the desired pH for further electrocatalytic experiments.

## Results

### Optimizing the Irradiation Parameters to Control the Size and Density of Au Nanoparticles

Metal nanostructures are highly rated and the primary choice for many applications such as catalysis, sensing, and smart and portable electronics and optoelectronics due to their intrinsic physicochemical properties, along with rapid improvements in their preparation methods. In order to minimize the efforts and costs in line to their applications, *in situ* fabrication of metal nanostructures on a suitable substrate/support with outright control over shape, size, and reproducibility should be targeted. Pulsed laser ablation of thin-metal films deposited on conductive support provides a robust platform toward nanostructured surfaces. Depending upon the applications sought, suitable conditions for size, shape, and distribution-controlled nanostructuration can be defined by adapting both the laser and material parameters. Thus, spatially ordered nanostructures ranging from particles, micro- to nano-bumps, nanojets or spikes can be produced (Reichenberger et al., 2019). Using them in electrochemistry requires designing a specific conductive support that ensures their stability in electrolytes. In this work, we prepared a 2-D array of AuNPs by inducing the molten phase dewetting process via nanosecond pulsed laser irradiation of Au deposited as thin films on ITO support. The laser pulse duration (10 ns) was much higher than the characteristic time (50 ps) to reach the equilibrium between hot electrons and lattice. Hence, it can be expected that the melt dynamics of the gold film would be the dominant process in its nanostructuring under nanosecond laser irradiation (Ivanov and Zhigilei, 2003; Ruffino et al., 2012). Three different thicknesses (named 20, 30, and 40 nm) of the Au films were deposited on ITO-coated glass slides by smart coater. By using AFM, the thicknesses of the gold films were precisely measured to be 19.6 ± 0.6, 30.7 ± 0.2, and 37.61 ± 0.4 for 20 nm, 30 nm, and 40 nm deposited Au films, respectively.

**Figure 1:**
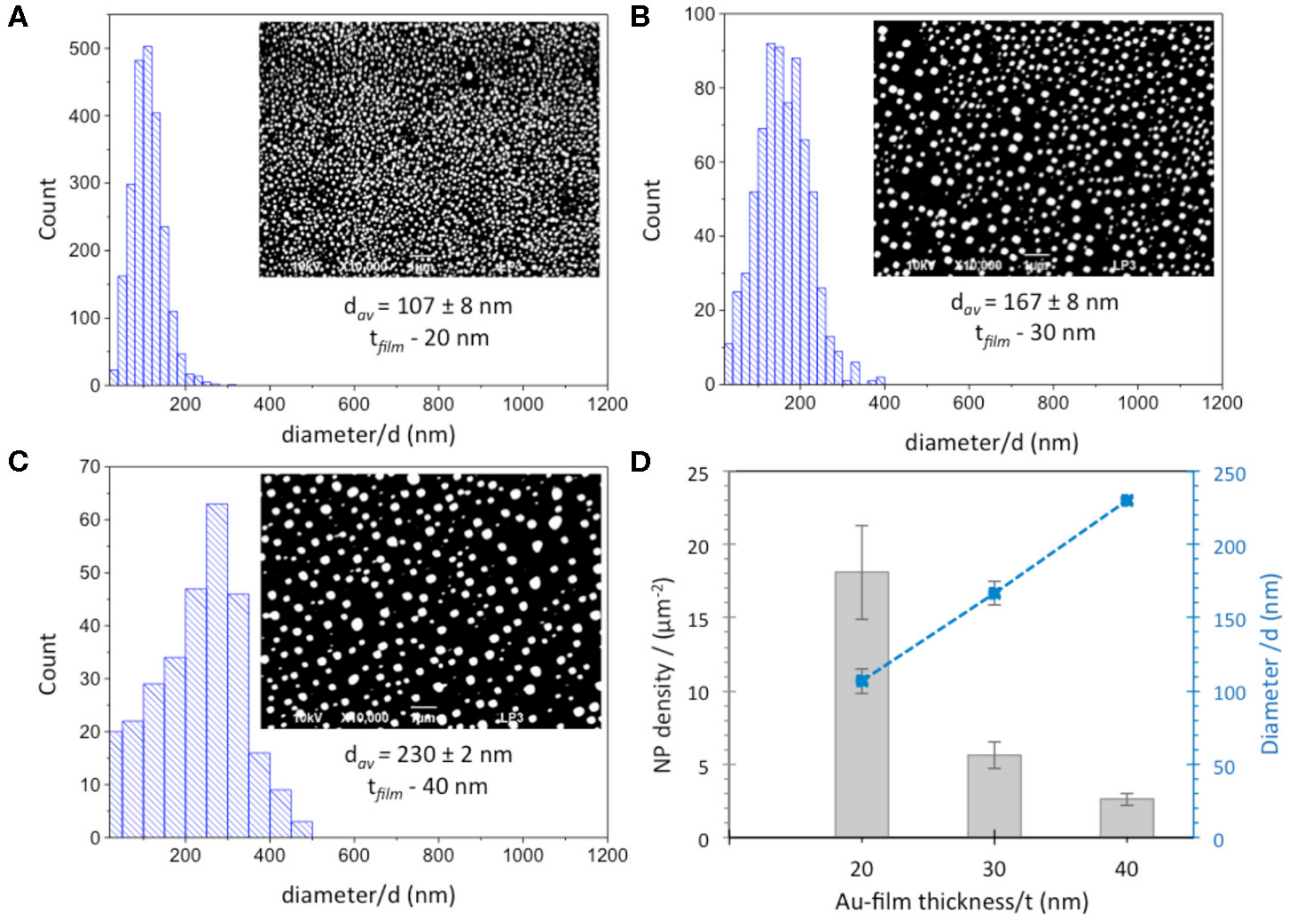
Size distribution and SEM images (inset) of AuNPs obtained from **(A)** 20 nm, **(B)** 30 nm and **(C)** 40 nm Au film thicknesses. Scale bar is 1 μm. **(D)** Dependence of diameter (blue squares) and density (gray columns) of AuNPs obtained after laser irradiation of Au-films on Au film thickness.

Thereafter, suitable parameters, such as laser fluence and the number of irradiation pulses, were optimized in order to pattern a homogeneous array of AuNPs. 20 and 30 nm films were irradiated by an energy density of 143 mJ/cm^2^ with 20 and 200 pulses, respectively, whereas optimized conditions used for 40 nm Au film thickness were 190 mJ/cm^2^ with 20 pulses. Figures 1A–C shows the size distribution of AuNPs and SEM micrographs (inset) obtained after optimization of irradiation conditions for the three different thicknesses of the Au films. The plot of the mean particle diameter, d, and their surface density as a function of the initial Au film thickness, t, is reported in Figure 1D. The density of the AuNPs increases with decreasing Au film thickness in the range (2.6 ± 0.4 – 18.6 ± 3.2 μm^−2^), while the mean particle diameter increases as the initial Au film thickness increases. The smallest NPs with an average diameter of ~100 nm were obtained by using the thinnest 20 nm Au film. Increasing the film thickness leads to the formation of comparatively larger NPs with average diameters of 167 ± 8 and 230 ± 2 nm for 30 nm and 40 nm Au film thicknesses, respectively, in agreement with previous works (Henley et al., 2005; Stankevicius et al., 2019). A broader range of nanoparticle size distribution was also observed for thicker films (30–40 nm) compared to the 20 nm Au film.

**Figure 2:**
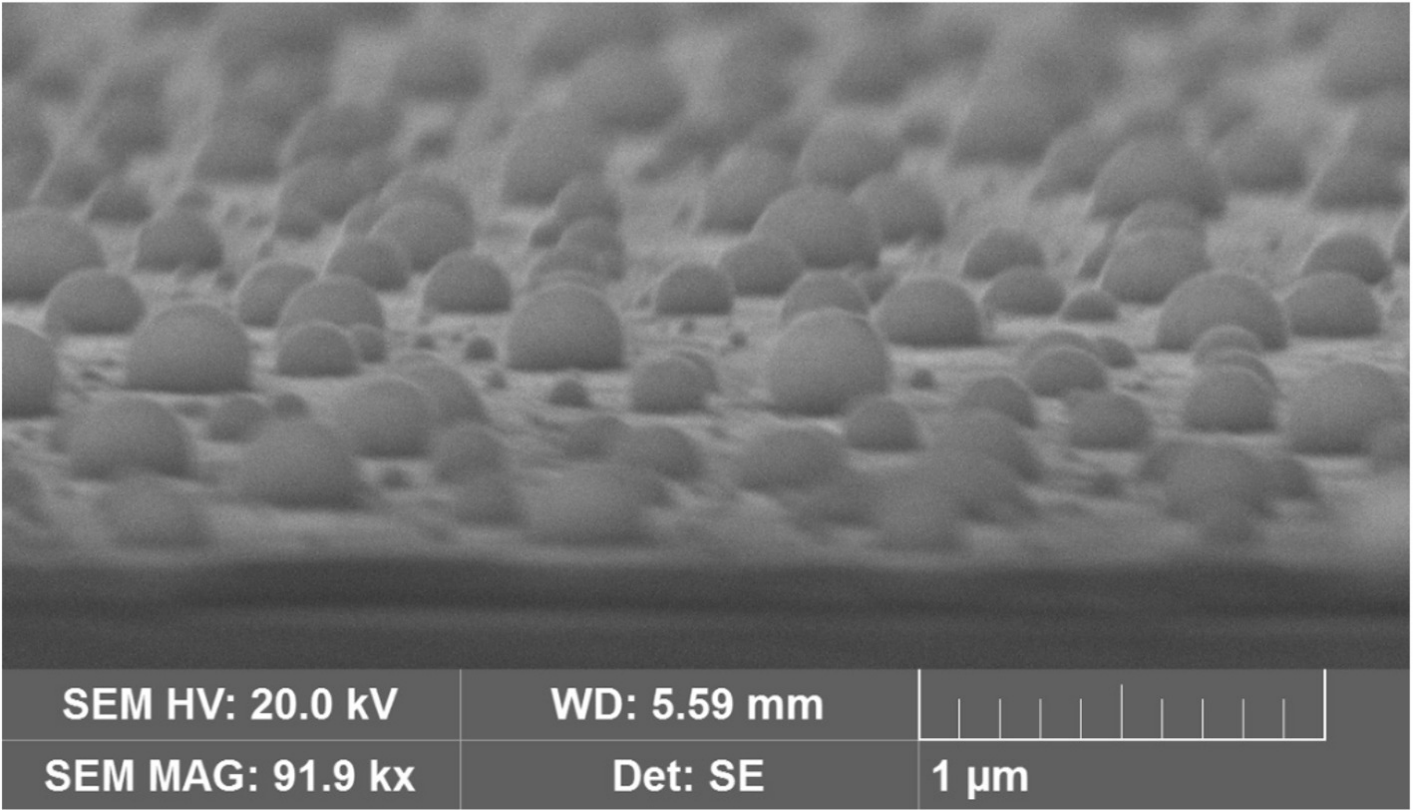
SEM images of AuNPs obtained from 40 nm Au film thickness after tilting the sample by 80°C.

Additional SEM experiments were made on AuNP@ITO samples to evaluate the contact angle of the AuNPs. It is shown that the average contact angle of the AuNPs is close to 90°. AuNPs are mainly hemispheres as observed previously for laser-treated thin metal films on SiO_2_/Si substrates (Henley et al., 2005) (Figure 2).

### Comparative Analysis of Geometric and Electroactive Surfaces Developed by AuNP@ITO Electrodes

**Figure 3:**
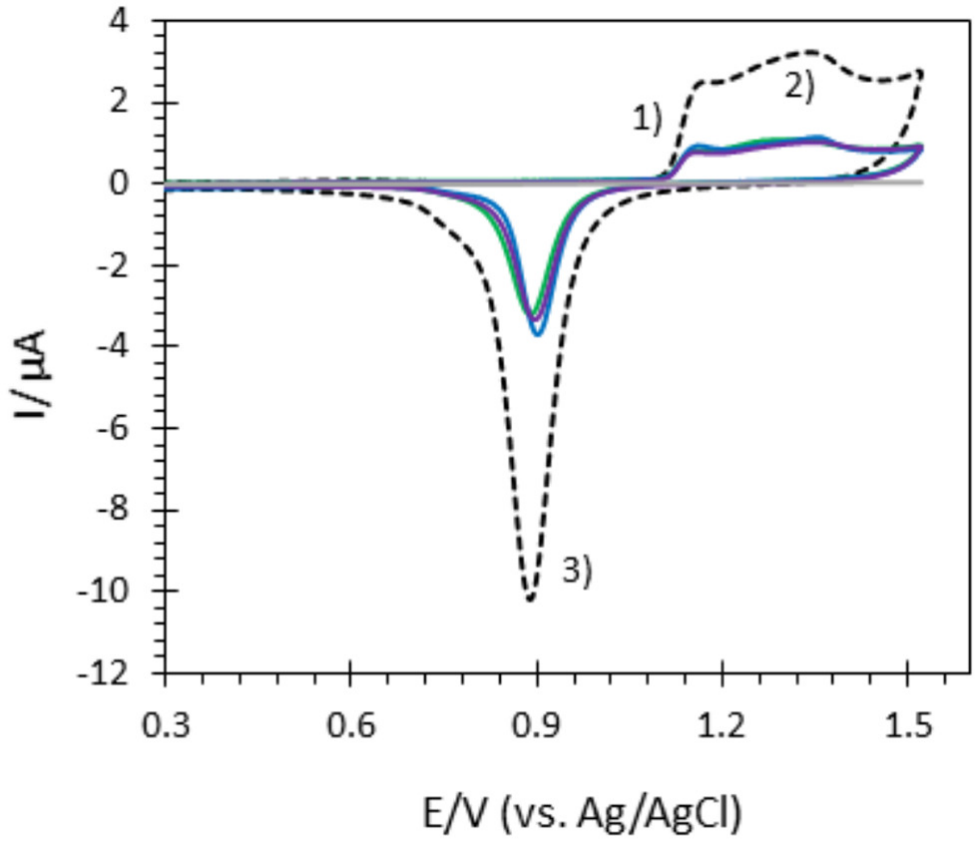
CVs of bare ITO (gray), Au-film@ITO (black dashed), and AuNP@ITO electrodes prepared from 20 nm (green), 30 nm (blue) and 40 nm (purple) Au films in 0.05M H_2_SO_4_. Scan rate 100 mV/s.

Several approaches have been carried out to evaluate the involvement of AuNPs in catalysis on the different AuNP@ITO electrodes compared to both the Au film surface (denoted Au-film@ITO) and to the bare ITO (Scheme 1 and Table 1). A_elect_ developed by AuNPs on an ITO electrode is calculated from the charge under the Au oxide peak at 0.9 V (peak 3 in the CV of Figure 3) observed by running typical cyclic voltammetry between 0.2 and 1.5 V vs. Ag/AgCl (see experimental section). The AuNP projected area (A_projected_/μm^2^) for the AuNP@ITO electrode was calculated using ImageJ software by considering a circular footprint of NP, which is realistic considering the SEM images in Figures 1, 2. A_projected_ calculation neglects the contribution of spherical NPs, and it is thus only an indication of NP surface coverage on the ITO surface. It is reported in Table 1 as a percentage of ITO coverage. A_projected_ will be used in the following to assign the contribution of different catalytic signals, i.e., for, ITO or AuNP@ITO electrodes. A_real_ is obtained by considering the hemispherical shape of AuNPs for different Au film thicknesses. Using ImageJ software, the evaluation of the AuNP diameter (2R) and corresponding number of particles of a given diameter (N) allows the calculation of the surface area of each AuNP size distribution (N × 2πR^2^). The sum of these surface areas for all size distribution gives the A_real_ for a given SEM image and is then corrected to the total electrode surface.

**Table 1:** Comparative electrode areas and volumes developed by AuNP@ITO and Au-film@ITO electrodes considering electrochemical, projected, or real Au surfaces and volumes developed on the total sample (S = 0.0078 cm^2^).

**Thickness**	**Diameter**	**A_**projected**_**	**A**_****elect****_		**A**_****real****_		**V**_****real****_	
**t nm**	**d nm**	**%**	**10**^****5****^ **μm**^****2****^		**10**^****5****^ **μm**^****2****^		**10**^****3****^ **μm**^****3****^	
			**AuNP**	**Au film**	**AuNP**	**Au film**	**AuNP**	**Au film**
20	107 ± 8	21 ± 2.1	8.8 ± 1.5	21 ± 2	1.6 ± 0.2	7.8	7.2 ± 1.2	15.6
30	167 ± 8	14.6 ± 0.5	8.3 ± 1.1		1.1 ± 0.05		8.2 ± 0.9	23.4
40	230 ± 2	12.5 ± 0.2	6.4 ± 0.9		0.97 ± 0.02		9.9 ± 0.4	31.2

From data in Figure 3 and Table 1, some relevant information can be put forward. As expected, the bare ITO shows no redox activity in H_2_SO_4_ (gray curve, Figure 3). Au-film@ITO or AuNP@ITO display the typical redox behavior of Au with well-defined peaks for oxidation (denoted as 1 and 2) and reduction of oxides at 0.9 V (denoted as 3) (Burke and Nugent, 1997; Hitaishi et al., 2018a). Such defined redox character irrespective of the NP size and the density of the AuNPs is observed. A_elect_ developed by the AuNPs shows no clear dependence on size and distribution of the NPs, however (Figure 3). This is contrary to the finding by Stankevicius et al., where NPs with sizes in the 100 nm range were not electroactive (Stankevicius et al., 2019), a behavior attributed to possible side products deposited during laser treatment. Discrepancy between the two studies could arise from the way by which electrodes are designed for electrochemistry.

A surprising feature is that the highest peak currents are observed for Au-film@ITO electrodes compared to AuNP@ITO electrodes. Since the peak current magnitude is related to the respective A_elect_, this implies that Au-film@ITO displays the highest Au surface area. Although a previous study concluded that the effect of roughness of gold surfaces did not control the ET of immobilized cytochrome c (Millo et al., 2009), surface characteristics at the microscale may be an explanation. Indeed, the surface morphology could be different between the thin film deposited by the smart coater and AuNPs formed as a result of melting and cooling. In our conditions, the roughness factor R_f_ was calculated as the ration between A_elect_ and A_real_ (R_f_ = A_elect_/A_real_). R_f_ of ~5.5 and ~6.6–7.5 are calculated for ~100 nm and ~150–230 nm AuNPs respectively, against ~2.7 for the Au-film surface. R_f_ values would indicate that an Au film is much smoother than an NP surface, which is unexpected for laser-obtained AuNPs.

It must be kept in mind that A_elect_ is an experimental value calculated electrochemically which is independent of any microscopy observations. However, experimental errors can be more significant in numerical calculations from SEM observations. By this method, we measured a coverage of ITO by the AuNPs (A_projected_) decreasing from 21 to 12.5% when the size of nanoparticles increases (Table 1). The high value of R_f_ we calculated strongly suggests that some small particles of Au might not have been taken into account from the microscopic observation that could contribute to A_real_.

We also compared the volume V_real_ developed by AuNPs and the volume corresponding to the Au film for each Au film thickness. As can be extracted from Table 1, only 50% of available Au remains as AuNPs. Some detachment of Au droplets from the surface during laser treatment procedure can explain both this low percentage of Au remaining and hence the decrease in A_elect_ after AuNP formation (Habenicht et al., 2005). Effectively as a consequence of dewetting, in order to form a local droplet, transportation of melted gold toward the center of the metallic structure leads to a vertical movement of the center-of-mass, which may induce the liquid droplet to leave the surface due to inertia.

### Stability of an AuNP@ITO Electrode

**Figure 4:**
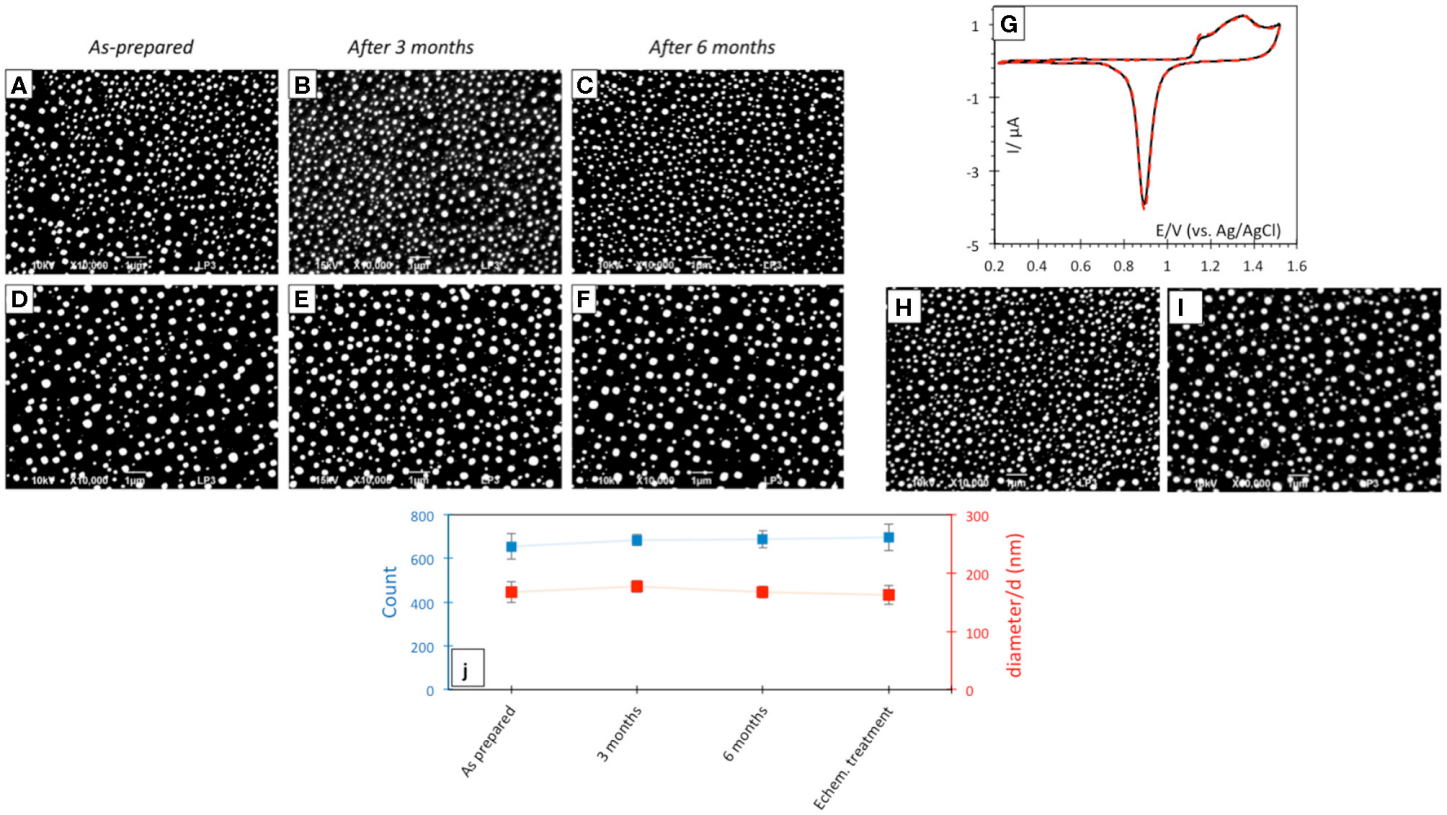
SEM micrographs after laser treatment of 30 **(A–C)** and 40 nm **(D–F)** Au film thicknesses respectively, before **(A,D)** and after 3 **(B,E)** or 6 **(C,F)** months storage at room temperature in air. **(G)** CVs in 50 mM H_2_SO_4_ of as prepared (black solid curve) and after 3 month storage (red dotted curve) AuNP@ITO sample from 30 nm Au film. Scan rate 100 mV/s. **(H,I)** SEM micrographs after electrochemical and chemical treatments of AuNPs from 30 nm **(H)** and 40 nm **(I)** Au films. **(J)** Count and size variation of AuNP@ITO samples from 30 nm Au film as prepared and after storage or electrochemical/chemical treatments.

Laser-induced AuNP@ITO electrodes were stored at room temperature and SEM micrographs realized before/during/after 6 months storage under air atmosphere were compared (Figure 4). No significant changes in the AuNP morphology, density, or size of the dots can be noticed after storage (Figures 4A–F). The effect of AuNP aging on the AuNP@ITO electrochemical signal was then evaluated (Figure 4G).

A similar CV response characteristic of Au redox behavior in acid was recorded from AuNP@ITO electrodes either as prepared or stored at RT in air for 3 months. The stability of AuNP@ITO electrodes after typical electrochemical treatment (CV in H_2_SO_4_ for cleaning and evaluation of the surface developed by the AuNPs), and further chemical treatments for chemical modification (especially thiol modification required for bioelectrocatalysis in the next steps) was then checked. SEM visualization and analysis of the AuNPs after these treatment steps show no significant changes in NP count or size of the AuNPs (Figures 4H,I). Figure 4J summarized the size and count of the AuNPs in the different conditions investigated, clearly highlighting the high stability of the laser-ablated AuNPs.

### Bioelectrocatalysis on AuNP@ITO Electrodes

**Figure 5:**
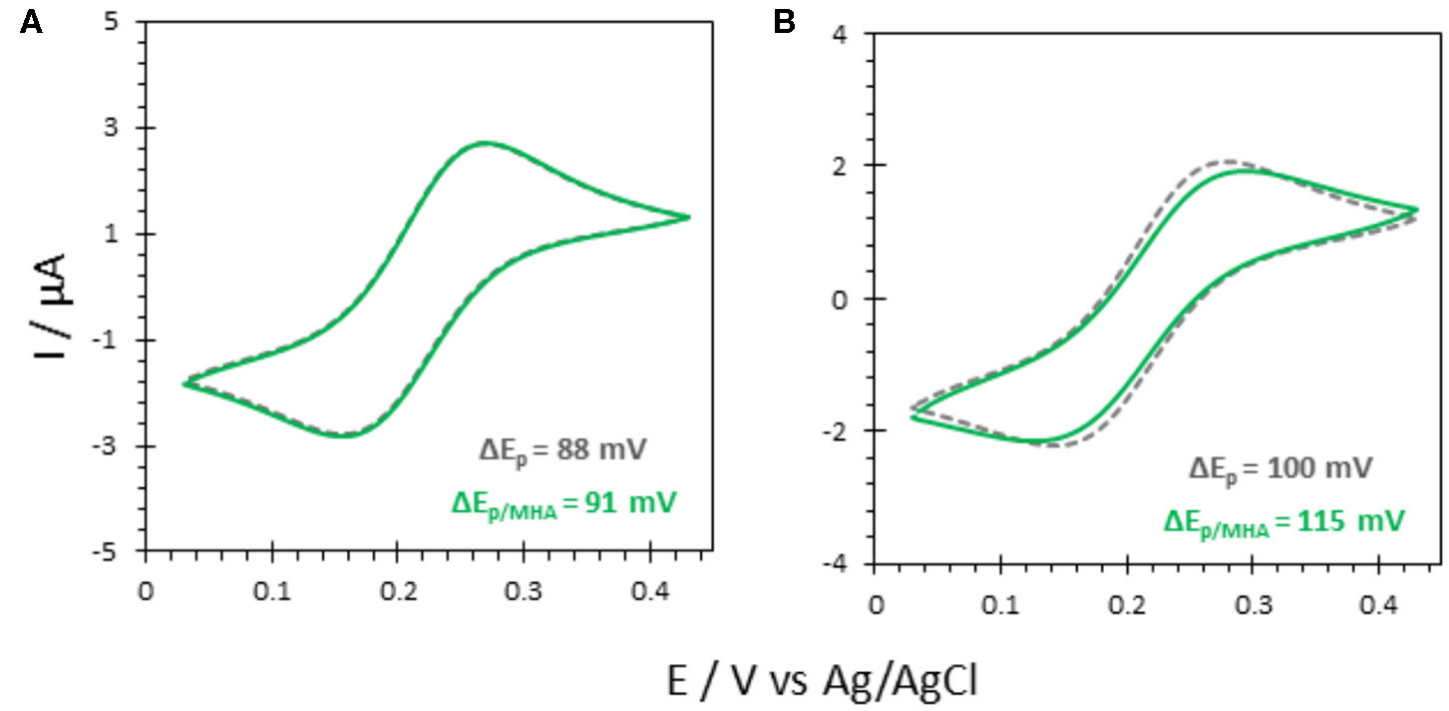
CV responses of 5 mM FeCN on **(A)** bare ITO and **(B)** AuNP@ITO (from 30 nm Au film thickness) before (gray curves) and after immersion in 6-MHA (green curves). Scan rate 20 mV/s. Phosphate buffer 100 mM, pH 6.

To investigate the effectiveness of AuNP@ITO electrodes for bioelectrocatalysis, the propensity of FeCN to behave as a reversible electrochemical system on these electrodes was first studied. The effect of modifying the ITO or AuNP surface with organic layers, such as self-assembled monolayers (SAMs) from thiol derivatives, was especially investigated (Figure 5).

Actually, a preferred adsorption of carboxylic acids functionalities over thiols on semiconductor oxide surfaces, such as ITO, was shown (Gardner et al., 1995; Yan et al., 2000). Multiscale spectroscopic, microscopic and electrochemical studies of preparation and characterization of SAMs on ITO also demonstrated a large percentage of thiols adsorbed as unbound molecules (Millo et al., 2009). In our conditions, the reversible redox process for FeCN was not affected by the immersion of the ITO electrode in a carboxylic end group thiol solution (6-MHA). Both peak currents and potentials remain unchanged (Figure 5A), suggesting that no 6-MHA adsorption on ITO happens in our conditions. When AuNP@ITO electrodes were modified by 6-MHA, a small increase in peak potential difference was observed for the redox signal of FeCN, that may be ascribed to selective thiol modification of AuNPs that slows down ET for FeCN (Figure 5B).

The two redox enzymes were then investigated once immobilized on AuNP@ITO, i.e., bilirubin oxidase (*Mv* BOD) from the fungus *Myrothecium verrucaria* and laccase from the bacterium *Thermus thermophilus* (*Tt* LAC). The molecular basis for the immobilization of these enzymes were previously determined in our lab (Hitaishi et al., 2018a, 2020). In correlation with values and directions of dipole moments, as well as charges surrounding the first electron acceptor, the Cu T1, we demonstrated that negative (respectively positive) charges on the electrode favored the direct electron transfer (DET) for O_2_ reduction with *Mv* BOD (resp. *Tt* LAC).

**Figure 6:**
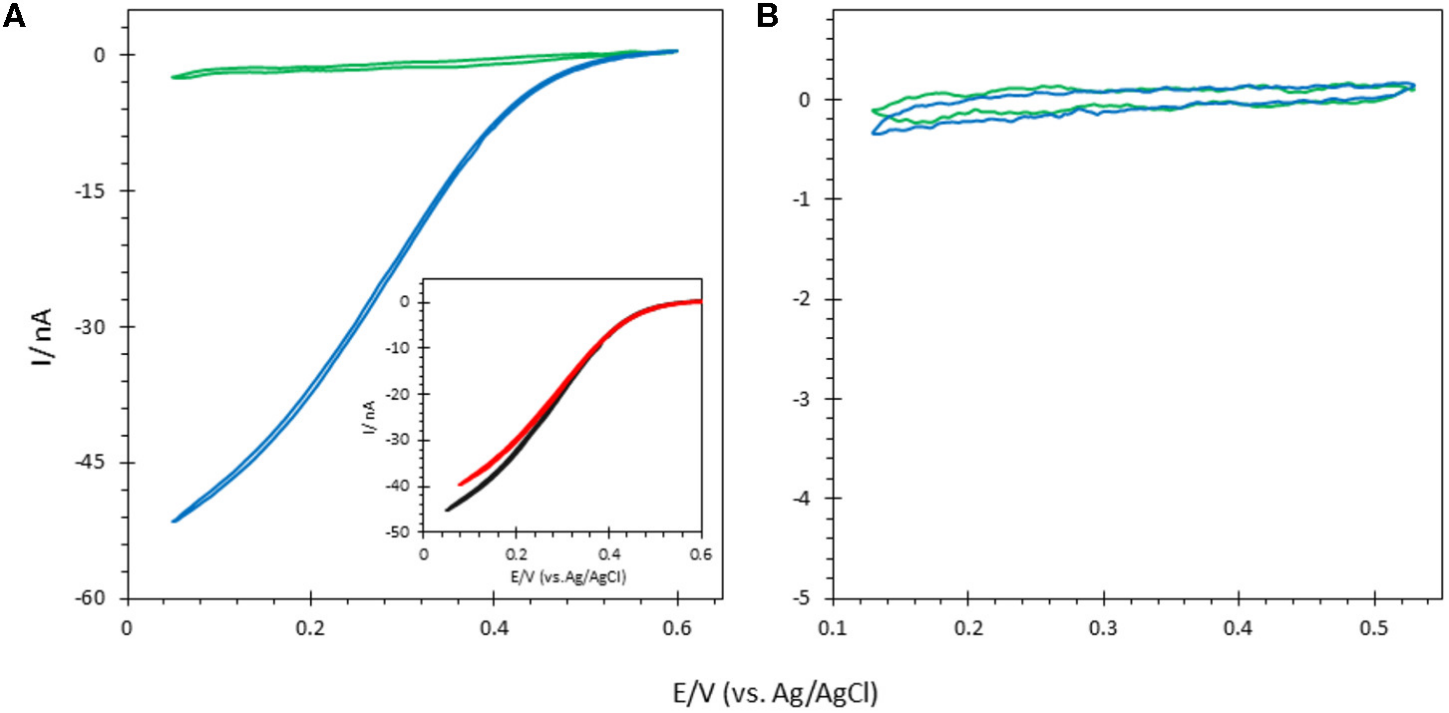
CVs obtained under N_2_ (green) and O_2_ (blue) after **(A)** Mv BOD and **(B)** Tt LAC adsorption on bare ITO electrode; Inset in **(A)** CV of Mv BOD adsorbed on ITO treated with 6-MHA (black) or CYST (red). Scan rate 10 mV/s. 100 mM phosphate buffer pH 6 for Mv BOD and acetate buffer pH 5 for Tt LAC. Enzyme adsorption is made at 4°C and electrochemistry is carried out at 25°C.

The elctroactivity of both enzymes on ITO electrodes was first investigated (Figure 6). The sigmoidal CV curve observed in the presence of O_2_ with *Mv* BOD adsorbed on ITO electrodes indicates that bioelectrocatalytic oxygen reduction occurs via DET. From the onset potential (550 mV vs. Ag/AgCl), it also suggests that adsorbed *Mv* BOD adopts an orientation in which the physiological electron entry site, the Cu T1, faces the ITO surface for electron exchange. *Mv* BOD adsorption in an electrocatalytically favorable orientation on ITO is most probably triggered by the hydrophilic negative nature of the ITO surface induced through cleaning by ultrasonication in Milli-Q water/ethanol (1:1) (Szot et al., 2013). The positive environment around the T1 Cu associated to a strong dipolar moment at pH 6 thus leads to an orientation favorable for ET, as previously defined (Hitaishi et al., 2018a; Gutierrez-Sanchez et al., 2019). On the other hand, no catalysis was observed when the bioelectrodes were prepared with *Tt* LAC. A favorable orientation is not adopted for the ET in this case as the net negative charge around T1 Cu (at pH 5) is repelled by the negative ITO surface (Hitaishi et al., 2018a, 2020).

**Figure 7:**
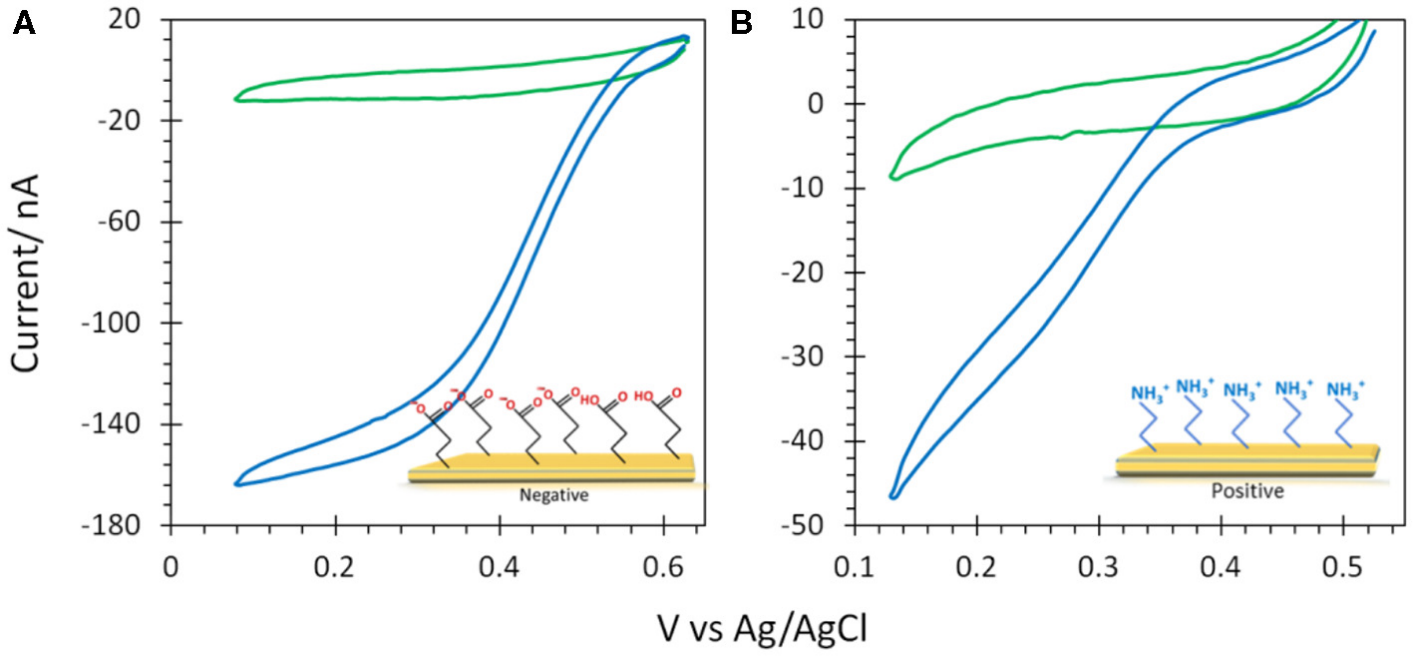
CVs obtained under N_2_ (green) and O_2_ (blue) after **(A)** *Mv* BOD adsorption on 6-MHA and **(B)** *Tt* LAC adsorption on CYST functionalized Au-film@ITO (30 nm) electrodes. Scan rate 10 mV/s, 100 mM phosphate buffer pH 6 for *Mv* BOD and acetate buffer pH 5 for *Tt* LAC. Enzyme adsorption was made at 4°C and electrochemistry is carried out at 25°C.

FeCN electrochemical study above suggested that thiol was not able to adsorb on the ITO. In order to further check the effect of thiol on bioelectrocatalysis on the ITO surface, the ITO electrode was traeted by 5 mM 6-MHA or CYST ethanolic thiol solution followed by *Mv* BOD adsorption. Whatever the thiol either 6-MHA or CYST, the treatment had no influence on biocatalysis in terms of current, onset of potential, and CV shape, again suggesting the absence of thiol layers on the ITO (Figure 6A). However, we confirmed the validity of the electrostatic model previously proposed by Hitaishi et al. with the enzymes adsorbed on Au-film@ITO modified by the suitable SAM previously demonstrated to allow DET, i.e., carboxylic-based (6-MHA) and amino-based (CYST) SAMs for *Mv* BOD and *Tt* LAC, respectively (Figure 7) (Hitaishi et al., 2018a). As expected, DET was obtained in each case with onset potentials of 560 and 420 mV vs. the Ag/AgCl characteristic of Cu T1 in *Mv* BOD and *Tt* LAC, respectively.

**Figure 8:**
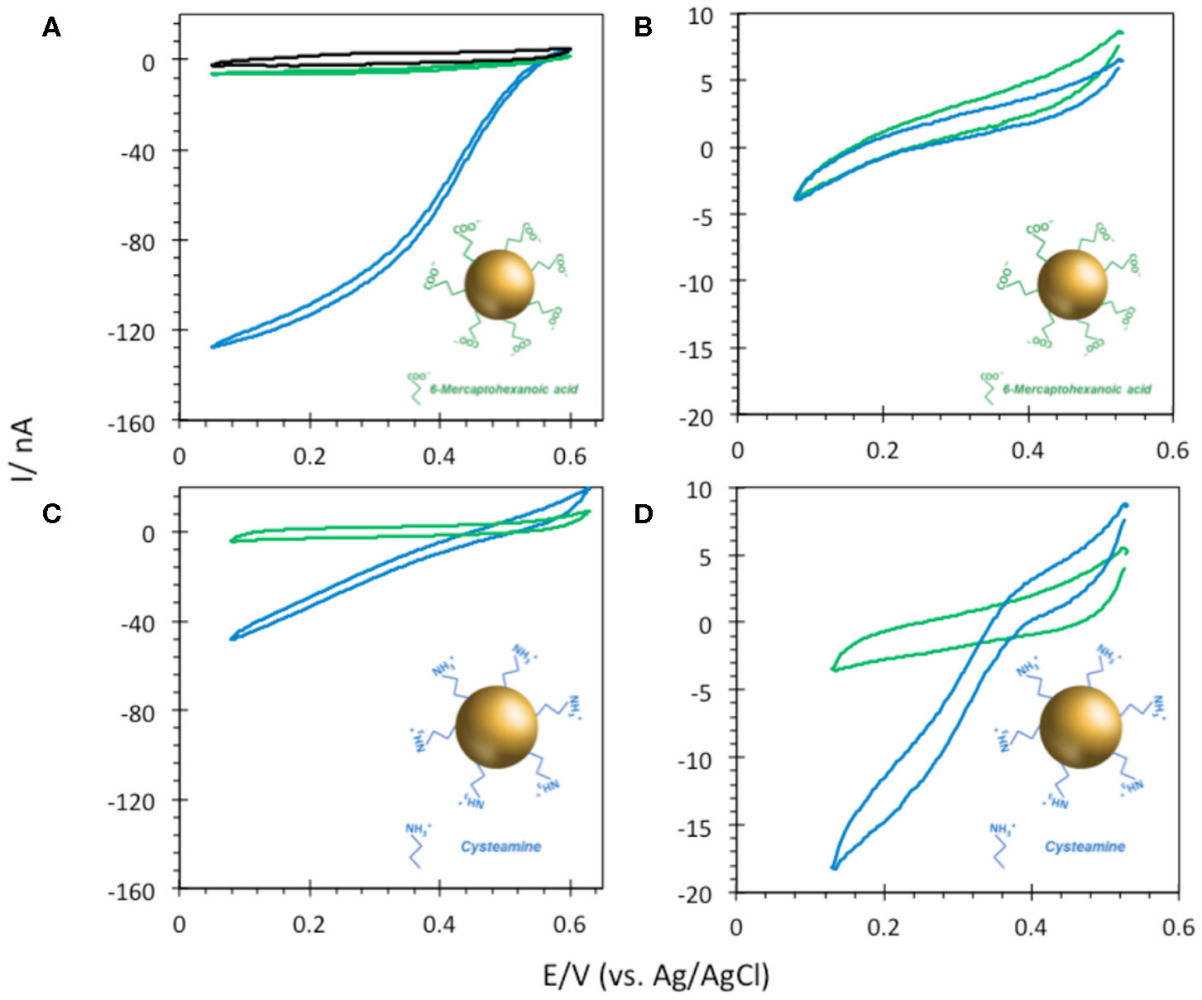
CVs obtained under N_2_ (green) and O_2_ (blue) on **(A,B)** 6-MHA and **(C,D)** CYST functionalized AuNP@ITO (from Au film thickness of 30 nm) electrodes with adsorption of Mv BOD **(A,C)** or Tt LAC **(B,D)**. In **(A)** the black curve corresponds to CV under O_2_ of AuNP@ITO in the absence of enzyme. Scan rate 10 mV/s, 100 mM phosphate buffer pH 6 for Mv BOD, and acetate buffer pH 5 for Tt LAC.

6-MHA and CYST thiols were finally used to provide negative and positive AuNP@ITO surfaces, respectively (Figure 8). We observe that negative 6-MHA functionalized AuNPs promote DET catalysis with *Mv* BOD (Figure 8A), whereas no catalysis occurs with *Tt* LAC (Figure 8B). An opposite catalytic behavior is obtained with *Mv* BOD and *Tt* LAC adsorption on positive CYST functionalized AuNPs (Figures 8C,D). The CV recorded with *Mv* BOD reflects weak catalysis on the remaining ITO surface, while DET is observed with *Tt* LAC. It is thus strongly suggested that AuNP functionalization drives the bioelectrocatalysis following the electrostatic model described for Au-film@ITO. The current magnitude of the catalytic signal was only weakly affected by the size of AuNPs (data not shown). Actually, it should be reminded that the size (100 to 230 nm of diameter) and density (2.6 to 18.6 μm^−2^) of the AuNPs are inversely correlated and that both are a function of gold film thickness. Therefore, visualizing the effect of AuNP size only on bioelectrocatalysis remains challenging.

**Figure 9:**
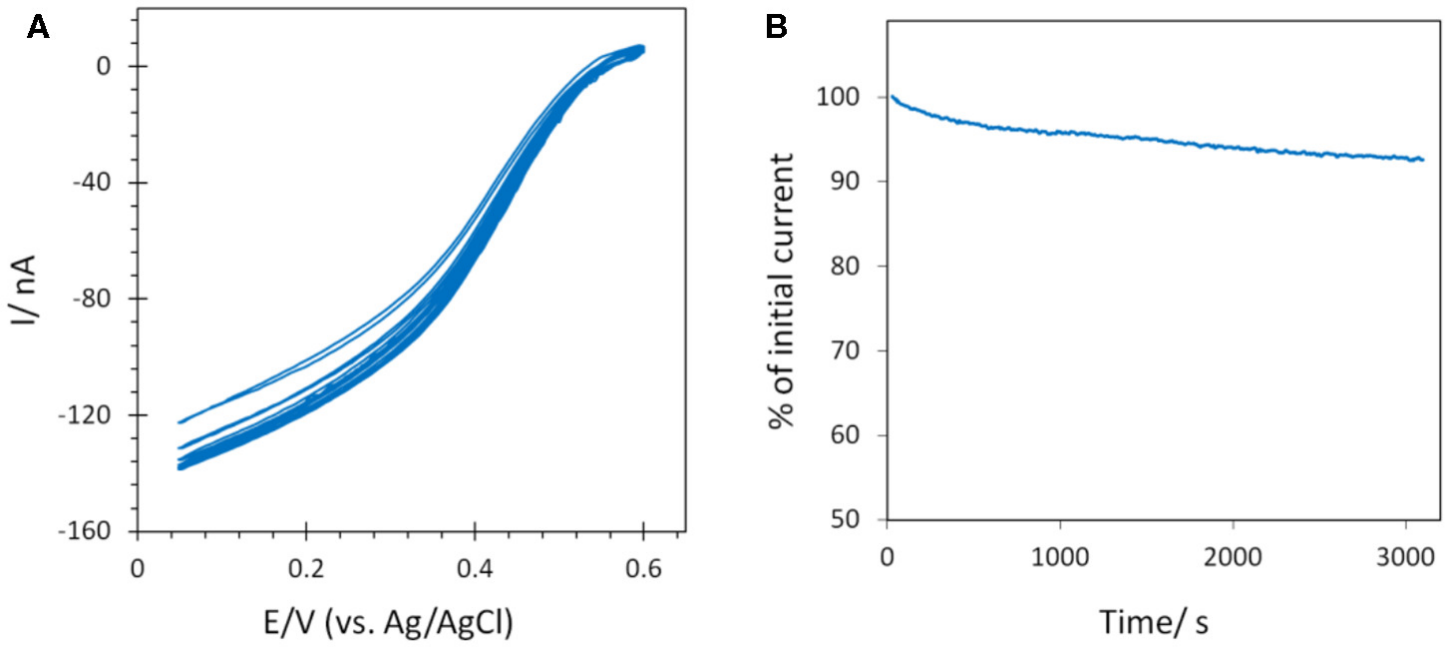
Stability of the catalytic signal by **(A)** CV continuous cycling (45 min) and **(B)** chronoamperometry (~50 min) after Mv BOD adsorption on 6-MHA AuNP@ITO (from Au film thickness of 30 nm) electrodes. Scan rate 10 mV/s for CV, and chronoamperometry was recorded at 0.1 V. 100 mM phosphate buffer pH 6.

The stability of the catalytic process obtained using *Mv* BOD adsorbed on 6-MHA modified AuNP@ITO electrodes was evaluated by continuous CV cycling and chronoamperometry (Figure 9). A small activation of the catalytic activity was observed for the first three CV cycles, and attributed to the rearrangement in the adsorbed enzyme layer under variable applied potential as observed in our previous study (Hitaishi et al., 2018a). After 45 min. of continuous cycling, a mere decrease (4%) in the catalytic current was recorded. Similarly, the application of a fixed potential of 0.1V vs. Ag/AgCl for a duration similar to the CV results in a very stable catalytic response. Only 6% of the initial activity was lost. A stable catalytic behavior on laser treated AuNPs is in good agreement with our previous study on polycrystalline Au electrodes (Hitaishi et al., 2018a), indicating that the laser method can not only be used to form controlled AuNP-based electrodes, but also that they can be applied for durable and efficient bioelectrocatalysis.

**Figure 10:**
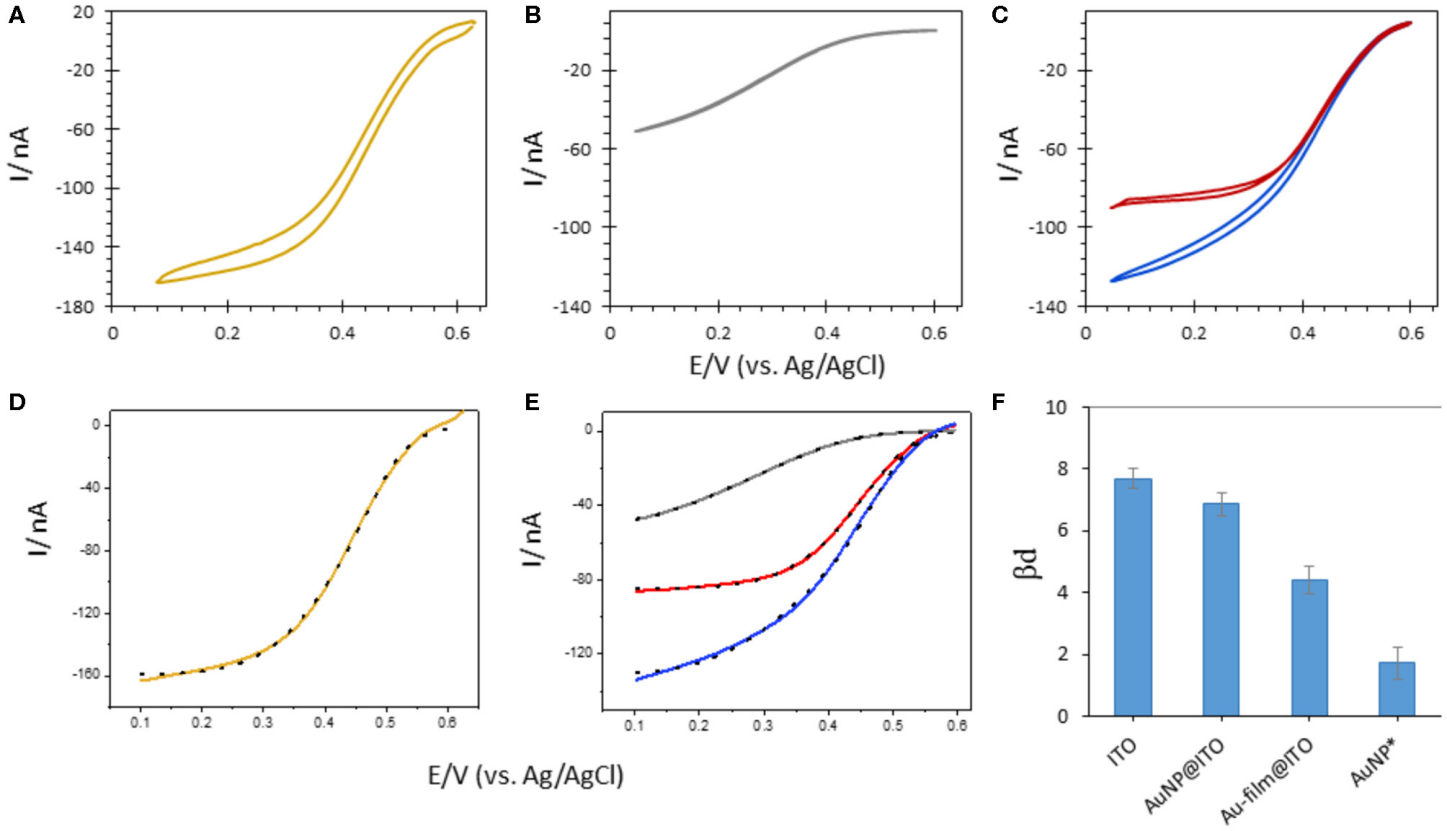
CVs obtained after Mv BOD adsorption on 6-MHA functionalized **(A)** Au-film@ITO **(B)** bare ITO or **(C)** AuNP@ITO (blue curve) electrodes. In **(C)** the red curve is obtained after subtraction of 86% ITO CV signal from AuNP@ITO one. **(D,E)** Electrocatalytic modeling (black dotted line) of corresponding CVs (solid line). **(F)** Changes in βd upon Mv BOD adsorption as a function of different electrode surfaces. AuNPs* represents the values for average subtracted CV (CV_AuNPs@ITO_ – CV_ITO86%_). Scan rate 10 mV/s, 100 mM phosphate buffer pH 6.

The next question is whether laser-made AuNPs could have an impact on the rate of the interfacial ET. In order to address the issue of orientation of *Mv* BOD immobilized on ITO electrodes with and without AuNPs, the fitting of electroenzymatic curves was made by following the formalism first developed by Armstrong and co-workers and applied to *Mv* BOD immobilized on carbon nanotube networks (Mazurenko et al., 2016) (Figure 10). Following the Marcus theory (Marcus and Sutin, 1985) for the biological ET, a distance-dependent constant β*d* called a “dispersion parameter” is defined, which results in a distribution of ET rates. The lower the β*d*, the narrowest the distribution of enzyme orientations is. Applied to BOD, d is the distance between the electron entry site of the immobilized enzyme, the T1 Cu, and the electrode surface.

The highest value of β*d* (7.67 ± 0.16) was calculated when *Mv* BOD was adsorbed on the ITO electrode. This value went along with the shape of the CV curve markedly different than the CV obtained for *Mv* BOD adsorption on Au-film@ITO. The β*d* value drops to 4.39 ± 0.22 for biocatalysis on the Au-film@ITO surface, suggesting a narrower distribution of ET rates and enzyme orientations. Hence, *Mv* BOD attained a more favorable orientation on a 6-MHA-functionalized Au-film@ITO surface due to well-defined electrostatic interactions. The β*d* value for the Au-film@ITO electrode is in close agreement with our previous studies on polycrystalline Au electrodes, where a β*d* value of 4.9 ± 0.19 was calculated at pH 6. Further immobilization of *Mv* BOD on 6-MHA modified AuNP@ITO electrodes gives a β*d* value of 6.86 ± 0.18, slightly lower than on bare ITO surfaces. This may indicate a weak shift toward narrow orientation distributions. However, since the AuNP@ITO electrode consists of both the AuNP and ITO surfaces, both responding to *Mv* BOD, a higher β*d* for AuNP@ITO than for Au-film@ITO surface may signify mixed distribution of enzyme orientations. To evaluate *Mv* BOD orientation on AuNPs only, we assume that the projected area (A_projected_ in Table 1) calculated from imageJ software is the area covered by AuNPs, and that rest of the surface is only ITO. Following this hypothesis, the CV responses on ITO alone were subtracted from CVs on AuNPs@ITO, considering the contribution of the ITO surface (i.e., 86% for 30 nm Au film thickness). The contribution of enzymatic catalysis on AuNP@ITO corrected from the contribution on ITO is given in Figure 10C, red curve. The modeling of this CV signal yields a very low β*d* value of 2.4 ± 0.4, indicative of a very narrow orientation distribution, never obtained in previous works.

Actually, enhanced bioelectrocatalysis, when using NP-based electrodes, is generally explained by the following three reasons. First, the increase in the surface-to-volume (S/V) ratio of a nanostructured electrode, which is inversely proportional to the NP radius, will promote high protein loadings, and thereby high current outputs, as the NP size decreases (Pankratov D. V. et al., 2014; Monsalve et al., 2015). Second, overpotentials are reduced on smaller NPs, probably due to a fast electron transfer between the electrode and the protein (Melin et al., 2013). Third, the spherical character of the NPs, which depends upon their synthetic strategy, plays an important role in the biocatalytic efficiency (Bastus et al., 2011). It was otherwise suggested that using AuNPs with a size comparable to the enzyme size, the distance between the nanoparticle and the active site of the enzyme should be decreased, inducing higher ET rates (Kizling et al., 2018). This is not the case here, since the AuNP size is ten times higher than the size of the enzyme. To explain the narrow distribution of enzymes, the arrangement of the thiol layer and/or the shape of the AuNPs should be evocated. A preferred adsorption of the enzyme molecules on the AuNPs edges, where their interaction with both Au and ITO surfaces drives a defined set of orientations, cannot be excluded either. Although this has to be investigated in depth in future works, this property appears as a great opportunity to yield efficient bioelectrocatalysis.

## Conclusions and Future Directions

Our previous studies on polycrystalline gold electrodes suggested that inter-enzyme interactions, either during their immobilization or during electrical communication on the electrode surface, could potentially influence the catalytic performance of the bioelectrode (Hitaishi et al., 2018a). Studying such interactions requires innovative strategies toward well-regulated enzyme-enzyme partition or enzyme nano-patterning on the electrode surface. In this work, we aimed at controlling the spatial organization/distribution of enzymes on the electrode surface by designing an ordered AuNP array via nanosecond laser irradiation of thin Au-films predeposited on ITO surfaces.

Such an obtained 2D array of AuNPs shows excellent stability, while offering the possibility of easy suitable functionalization. Two enzymes from the MCO family, namely *Mv* BOD and *Tt* LAC, showed opposite surface sensitivities on a given chemistry of thiol-based functionalized NPs, thus highlighting the viability of the electrostatic model previously proposed. Combining the advantages of AuNPs and thiol chemistry, a narrow distribution of enzyme orientations on laser-induced AuNPs was obtained for efficient electrocatalysis. However, NP size and density are changing simultaneously along with the Au film thickness. Therefore, while higher biocatalytic current outputs are expected with smaller AuNP sizes, studying the effect of NP size on biocatalysis is not trivial. This will be investigated in the close future by depositing the Au film on another substrate than ITO, which will allow increasing the laser energy, hence decreasing the thickness of the Au film to further decrease the size of AuNPs. In addition, several other structures can be developed using a laser with a defined shape and morphology that could be helpful in bioelectrochemical studies.

## Data Availability Statement

The raw data supporting the conclusions of this article will be made available by the authors, without undue reservation.

## Author Contributions

VH carried out the electrochemical experiments, laser treatment of NPs and analysis of NP by microscopy with AV. IM performed the SEM images of the tilted samples. AV carried out AFM measurements. GC initiated the construction of laser setup. VH wrote the initial version of the result section. EL wrote the full manuscript. All authors participated in the discussion of results and edition of the final version of the manuscript. PD and EL initiated the project and supervised the experiments.

## Conflict of Interest

The authors declare that the research was conducted in the absence of any commercial or financial relationships that could be construed as a potential conflict of interest.
